# Food applications of natural antimicrobial compounds

**DOI:** 10.3389/fmicb.2012.00287

**Published:** 2012-08-08

**Authors:** Annalisa Lucera, Cristina Costa, Amalia Conte, Matteo A. Del Nobile

**Affiliations:** Lab of Food Processing and Packaging, Department of Food Science, Agricultural Faculty, University of FoggiaFoggia, Italy

**Keywords:** antimicrobial compounds, food preservation, natural compounds, essential oils, shelf-life extension

## Abstract

In agreement with the current trend of giving value to natural and renewable resources, the use of natural antimicrobial compounds, particularly in food and biomedical applications, becomes very frequent. The direct addition of natural compounds to food is the most common method of application, even if numerous efforts have been made to find alternative solutions to the aim of avoiding undesirable inactivation. Dipping, spraying, and coating treatment of food with active solutions are currently applied to product prior to packaging as valid options. The aim of the current work is to give an overview on the use of natural compounds in food sector. In particular, the review will gather numerous case-studies of meat, fish, dairy products, minimally processed fruit and vegetables, and cereal-based products where these compounds found application.

## Introduction

Many food products are perishable by nature and require protection from spoilage during their preparation, storage, and distribution to give them desired shelf life. The demand for minimally processed, easily prepared, and ready-to-eat fresh food products, globalization of food trade, and distribution from centralized processing pose major challenges for food safety and quality. Food products can be subjected to contamination by bacteria and fungi. Many of these microorganisms can cause undesirable reactions that deteriorate flavor, odor, color, sensory, and textural properties of foods. Microbial growth is a major concern because some microorganisms can potentially cause food-borne illness. In packaged foods, growth and survival of common spoilage and pathogenic microorganisms such as *Listeria monocytogenes, Escherichia coli* O157, *Salmonella, Staphylococcus aureus, Bacillus cereus, Campylobacter, Clostridium perfringens, Aspergillus niger*, and *Saccharomyces cerevisiae* are affected by a variety of intrinsic factors, such as pH and presence of oxygen or by extrinsic factors associated with storage conditions, including temperature, time, and relative humidity (Singh et al., 2003; Lòpez-Malo et al., 2005; Rydlo et al., 2006).

To prevent growth of spoilage and pathogenic microorganisms in foods, several preservation techniques, such as heat treatment, salting, acidification, and drying have been used in the food industry (Davidson and Taylor, 2007; Farkas, 2007). Numerous efforts are conducted to find natural alternatives to prevent bacterial and fungal growth in foods. In recent years, because of the great consumer awareness and concern regarding synthetic chemical additives, foods preserved with natural additives have become very popular. To inhibit growth of undesirable microorganisms in food, the antimicrobials can be directly added into the product formulation, coated on its surface or incorporated into the packaging material. Direct incorporation of active agents into food results in an immediate but short-term reduction of bacterial populations, while the antimicrobial films can maintain their activity for a long period of time (Appendini and Hotchkiss, 2002; Hanušová et al., 2009).

Main natural compounds are essential oils derived from plants (e.g., basil, thyme, oregano, cinnamon, clove, and rosemary), enzymes obtained from animal sources (e.g., lysozyme, lactoferrin), bacteriocins from microbial sources (nisin, natamycin), organic acids (e.g., sorbic, propionic, citric acid) and naturally occurring polymers (chitosan). In this context, plant essential oils are gaining a wide interest in food industry for their potential as decontaminating agents, as they are Generally Recognized as Safe (GRAS). The active components are commonly found in the essential oil fractions and it is well established that most of them have a wide spectrum of antimicrobial activity, against food-borne pathogens and spoilage bacteria (Gutierrez et al., 2008, 2009). The antimicrobial activity of plant essential oils is due to their chemical structure, in particular to the presence of hydrophilic functional groups, such as hydroxyl groups of phenolic components and/or lipophilicity of some essential oil components (Dorman and Deans, 2000). Usually, the compounds with phenolic groups as oils of clove, oregano, rosemary, thyme, sage, and vanillin are the most effective (Skandamis et al., 2002). They are more inhibitory against Gram-positive than Gram-negative bacteria (Mangena and Muyima, 1999; Marino et al., 2001).

Allyl-isothiocyanate is the major antimicrobial component of mustard and horseradish oil. It has been found to be more effective against Gram-negative bacteria with less or no effect on lactic acid bacteria. Although its antimicrobial activity varies widely (Delaquis and Mazza, 1995), the volatile compound particularly inhibits *E. coli* (Nadarajah et al., 2002; Muthukumarasamy et al., 2003).

The use of bacteriocin-producing lactic acid bacteria or their more or less purified bacteriocins has been also receiving increased interest. Bacteriocins are small bacterial peptides that show strong antimicrobial activity against closely related bacteria. Nisin is a polypeptide produced by *Lactococcus lactis* spp. It has been approved as a food additive with GRAS status in over 50 countries worldwide. It has a relatively broad spectrum of activity against various lactic acid bacteria and other Gram-positive bacteria. Moreover, it is particularly effective against heat-resistant bacterial spores of *Clostridium botulinum* and against food-borne pathogens such as *L. mococytogenes, S. aureus*, or *B. cereus* (Brewer et al., 2002; Lopez-Pedemonte et al., 2003; Sobrino-Lopez and Martin-Belloso, 2006). Use of nisin in conjunction with ethylenediamine tetra-acetic acid (EDTA) may increase the effectiveness. Moreover, the effect of nisin can be enhanced by using exposure to chelating agents, sub-lethal heat, osmotic shock and freezing, because these treatments make the cell wall of Gram-negative microorganisms more permeable and therefore more susceptible to the nisin (Gálvez et al., 2007).

The enzymes represent another group of natural compounds that found application in food as valid preservatives. Lysozyme for example, is a lytic enzyme found in foods, such as milk and eggs, which can hydrolyze β-1,4 linkages between N-acetylmuramic acid and N-acetylglucosamin (Cunningham et al., 1991). Commercially, lysozyme has been used primarily to prevent late blowing in semi-hard cheeses, caused by *Clostridium tyrobutyricum*. It is well known that lysozyme is bactericidal against Gram-positive bacteria, whereas it is essentially ineffective against Gram-negative bacteria, owing to the presence of a lipopolysaccharide layer in the outer membrane. It has been recognized since the 1960's that susceptibility of Gram negative bacteria to lysis by lysozyme can be increased by the use of membrane disrupting agents, such as detergents and chelators (EDTA) (Vaara, 1992; Shelef and Seiter, 1993; Branen and Davidson, 2004).

Organic acids and their salts are widely used as chemical antimicrobial agents because their efficacy is generally well understood and cost effective. The most effective organic compounds are acetic, lactic, propionic, sorbic, and benzoic acid. Their antimicrobial effect is based on the increase in proton concentration thereby lowering the external pH. Organic acids may affect the integrity of microbial cell membrane or cell macromolecules or interfere with nutrient transport and energy metabolism, causing bactericidal effect (Ricke, 2003). Production of organic acids had been possible before the discovery of microorganisms, with lactic acid first being commercially produced by fermentation in 1880, but the majority of the organic acids produced were being chemically extracted or synthesized from other chemicals. Mixtures of acids could exert a wider antimicrobial activity than a single organic acid (Theron et al., 2010).

Among the natural antimicrobials, chitosan also received considerable interest for commercial applications. It has been used in medical, food, agricultural, and chemical industry, mainly due to its high biodegradability and antimicrobial properties. The biological activity of chitosan depends on its molecular weight, degree of deacetylation and derivatisation, such as degree of substitution, length, and position of a substitute in glucosamine units of chitosan, pH of chitosan solution and the target organisms (No et al., 2007). It is commercially produced from crab and shrimp shell wastes, with different deacetylation grades and molecular weights and, hence, it possess different functional properties, like emulsification ability, dye binding, and gelation. Chitosan has also been documented to possess a film-forming property for use as edible film or coating, to decrease water vapor and oxygen transmission, diminish respiration rate and increase shelf life of fruit (Jiang and Li, 2001).

This review focuses on the use of natural compounds to control microbiological and physicochemical shelf life of main food categories, such as meat, fish, dairy products, minimally processed fruit and vegetables and cereal-based goods. The information is mostly based on case-studies dealing with application of active compounds to prevent microbial proliferation occurring in packaged food during storage.

## Minimally processed food and vegetables juices

Minimally processed products are one of the major growing segments in food retail establishments. However, fresh-cut fruit and vegetables are widely studied because of the difficulties in preserving their fresh-like quality during prolonged periods. The goal of fresh-cut products is to deliver convenience and high quality. Taking into account the pressure of consumers about the use of synthetic chemicals, natural compounds have been suggested as a valid preservation technique (Table 1). Dipping, impregnation, coating, and spraying are the different ways of applications of active agents to fresh-cut fruit and vegetables but among them, the most recent results on the application of active compounds to ready-to-eat fruit and vegetables deal with coating systems. In the follow, some relevant examples are reported.

**Table 1:** **Relevant examples of natural active agents applied to minimally processed products**.

**Products and storage conditions**	**Natural compounds**	**Main results**	**References**
Fresh-cut papaya stored in trays at 4°C	A microencapsulated beta-cyclodextrin and trans-cinnamaldehyde complex (2 g/100 g) incorporated into a multilayered edible coating made of chitosan and pectin	The coating improved the microbiological and physicochemical quality of fresh-cut papaya. It extended the shelf-life up to 15 days compared to the control (<7 days)	Brasil et al., 2012
Cabbage packaged under vacuum, air and two MAP (100% N_2_ and 100% CO_2_)	Dipping treatment with acetic, lactic, and malic acids (1% and 2%)	Some pathogens inoculated on cabbage were significantly reduced by treatment with 2% acetic, 1% lactic, and 2% malic acids	Bae et al., 2011
Minimally processed broccoli packaged in multilayered polyolefin bags and stored at 5°C for 18 days	Edible coating based on chitosan and carboxymethyl-cellulose	The coatings retard product weight loss, browning, and yellowing, reduced stem hardening, microbial growth, and improved total chlorophyll and ascorbic acid retention	Ansorena et al., 2011
Ready-to-eat lettuce and carrots packaged in oriented polypropylene bags and stored at 4°C	Dipping treatment with oregano and thyme	The solution containing oregano recorded a significantly lower initial total viable count level than the water treatment on carrots. The sensory panel found essential oil treatments acceptable for carrots throughout storage, but no for lettuce rejected for overall appreciation by day 7	Gutierrez et al., 2009
Strawberries packaged under passive and active MAP with high (80% O_2_, 20% CO_2_) and low (65% N_2_, 30% CO_2_, 5% O_2_) percentage of oxygen	Solution of 1% chitosan	The chitosan coating inhibited growth of microorganisms and significantly affected the microbiological stability of the strawberries, above all when the samples were packaged under active MAP	Campaniello et al., 2008
Fresh-cut mushrooms packed in polyethylene bags and then stored at 4°C	Coating containing 5, 10, and 20 g of chitosan/L	At 4°C for 15 days, 20 g/L chitosan coating inhibited growth of total bacteria, yeasts, and moulds	Hesham, 2008

Malic acid in combination with various stabilizing compounds was used by Raybaudi-Massilia et al. (2009) for fresh-cut apples. As reported by Bico et al. (2009), the combined effect of chemical dip and/or edible coating and/or controlled atmosphere (CA) on quality of fresh-cut banana was investigated. Banana slices were dipped into a solution containing 1% (w/v) calcium chloride, 0.75% (w/v) ascorbic acid, and 0.75% (w/v) cysteine and/or combined with a carrageenan coating and/or combined with CA (3% O_2_ + 10% CO_2_). Dipping combined with CA treatment prevented product weight loss and increased polyphenol oxidase activity; regarding microbiological quality, the combined strategies prevented microbial growth after 5 days of storage at 5°C. The antimicrobial effects of propionic, acetic, lactic, malic, and citric acid against *E. coli* O157:H7, *S.* Typhimurium, and *L. monocytogenes* on whole red organic apples and lettuce were also clearly demonstrated by Park et al. (2011).

Raybaudi-Massilia et al. (2008) investigated the combined effects of malic acid and essential oils of cinnamon, palmarosa, and lemongrass (0.3 and 0.7%) and their main active compounds (eugenol, geraniol, and citral, 0.5%) on microbiological and physicochemical shelf-life of fresh-cut “Piel de Sapo” melon (*Cucumis melo L*.). The active compounds were incorporated into an alginate-based edible coating. Melon pieces were inoculated with a *S*. Enteritidis (10^8^ CFU/ml) culture before applying the coating. The incorporation of essential oils or their active compounds into the edible coating prolonged the microbiological shelf-life by more than 21 days. Pure citral and citron essential oil were added in the syrup of industrial ready-to-eat fruit salads stored at 9°C. Both citral (25–125 ppm) and citron essential oil (300, 600, 900 ppm) were able to prolong the microbial shelf-life. Citron essential oil doubled the time needed for the wild microflora to reach concentrations able to produce a perceivable spoilage in condition of thermal abuse (9°C). The same essential oil showed a strong inhibition against *L. monocytogenes*, but exerted limited effects on the survival of *S*. Enteritidis and *E. coli* (Belletti et al., 2008). Generally, when applying bioactive coatings containing essential oils to fruits and vegetables, one of the limiting factors is the impact of such components on the sensory characteristics of the coated products, mainly due to the great amount of volatile compounds which mask the natural flavor of fruits and vegetables. The use of compatible essential oil-foodstuff could also be a good alternative.

Natural volatile compounds such as methyl jasmonate, ethanol, tea tree oil, and garlic oil were applied on fresh-cut tomato stored at 5°C for 15 days. Ethanol combined with methyl jasmonate was more effective in suppressing microbial proliferation than each single compound. In addition, this combination preserved firmness and color better than the other antimicrobial preservatives. Moreover, methyl jasmonate let keep higher content of lycopene, ascorbic acid and phenolic compounds (Ayala-Zavala et al., 2008). Rojas-Graü et al. (2007) investigated the effect of lemongrass, oregano oil, and vanillin incorporated in apple puree-alginate edible coating, on the shelf-life of fresh-cut “Fuji” apples. During 21 days of storage at 4°C, the coating with vanillin (0.3% w/w) was the most effective in terms of sensory quality. All the other studied antimicrobial coatings significantly inhibited growth of psychrophilic aerobes, yeasts, and moulds. The antimicrobial effect of essential oils against *L. innocua* inoculated into apple pieces before coating was also tested. A release system of antimicrobial volatiles was adopted by encapsulation of garlic oil in β-cyclodextrin and tested on microbial growth and sensory quality of fresh-cut tomato (Ayala-Zavala and González-Aguilar, 2010). Grape-fruit seed extract was used as antimicrobial compound into a coating of sodium alginate to prolong the shelf-life of minimally processed kiwifruits. The combination of an active compound to an alginate-based coating delayed microbial growth, whereas the sole dipping treatment was inefficient. The combined use of modified atmosphere packaging (MAP) and coating treatments further prolonged the shelf-life up to 13 days (Mastromatteo et al., 2011a).

As reported by Krasaekoopt and Mabumrung (2008), the effectiveness of chitosan incorporated in the edible methyl cellulose coating on the microbiological quality of fresh-cut cantaloupe was evaluated. During storage at 10°C for 15 days, applications of 1.5 and 2% chitosan in the coating reduced growth of some pathogens, mesophilic aerobic bacteria (3.3 log cfu g^−1^), psychrotrophs (3.9 log cfu g^−1^), lactic acid bacteria (3.1 log cfu g^−1^), yeasts and molds (1.1 log cfu g^−1^), and total coliforms (3.8 log cfu g^−1^). An edible coating containing chitosan was also applied on carrot sticks to maintain quality and prolong the shelf-life (Simões et al., 2009). The coating application preserved the overall visual quality, the microbial proliferation and reduced surface whiteness during storage. While the content of total phenolics markedly increased in coated carrot sticks stored under moderate gas levels, it was controlled under low O_2_ and high CO_2_ levels. Film based on methyl cellulose incorporating chitosan and chitosan/methyl cellulose film incorporating vanillin were applied to fresh-cut cantaloupe and pineapple to control microbial quality during storage at 10°C (Sangsuwan et al., 2008). Chitosan/methyl cellulose film and vanillin films provided an inhibitory effect against *E. coli* in fresh-cut cantaloupe. The chitosan/methyl cellulose film rapidly reduced the number of *S. cerevisiae* inoculated on the products. For fresh-cut pineapples coating with vanillin was more efficient than chitosan/methyl cellulose in reducing the number of yeasts by 4 logs in six days. An edible coating with soy or wheat gluten protein as a carrier of thymol and calcium chloride was applied on strawberry by Atress et al. (2010). Treating fruits did not exhibit any change in fruit appearance until 9 days of storage. All treatments maintained ascorbic acid content, firmness, total sugar and reduced the total colony, moulds and yeasts compared to the control. The effect of sodium hypochlorite, peroxiacetic acid, acidified sodium chlorite and carvacrol on microbiological, sensory, and nutritional quality of fresh-cut jalapeno peppers stored at 5°C during 27 days was evaluated (Ruiz-Cruz et al., 2010). All sanitizers (except carvacrol) maintained microbiological and overall quality of jalapeno peppers during 27 days. Carvacrol, active ingredient of oregano essential oil, maintained shelf life for only 17 days and reduced sensory acceptability of fresh-cut produce. However, carvacrol-treated samples retained the highest levels of photochemical and antioxidant capacity.

Juices are food very susceptible to yeasts attack. *Pichia anomala, S. cerevisiae*, and *Schizosaccharomyces pombe* caused the most diffuse problems. Generally, heat treatment (pasteurization), aseptic packaging or use of weak acids exclude yeast spoilage. As alternative to these traditional artificial preservatives the use of natural compounds was proposed in the literature (Valero and Salmerón, 2003; Belletti et al., 2010). Clary, sage, juniper, lemon, and majoram essential oil were chosen to preserve apple juice, as being efficient in *in vitro* test and not containing phenolics but alcohol terpenoids, linalool (clary sage), and terpinen-4-ol (marjoram), the cyclic monoterpenes α-pinene (juniper) and limonene (lemon) (Tserennadmid et al., 2011). The anti-yeast effects of these essential oils were good in the acidic pH range optimal for yeasts growth. Synergism or additive effects were recorded by combining the different active compounds. The most interesting result of the study of Tserennadmid et al. (2011) was recorded with lemon essential oil. In fact, experiments with lemon given to apple juices showed that the “open” storage time at ambient temperature could be prolonged and a novel, refreshing taste could be achieved.

Although the influence of smell-taste of some active agents is known, it has not often been evaluated to a sufficient degree. One solution to the above-mentioned problem may be the use of combinations of different food preservation systems that would give the benefits of each of them while at the same time appreciably reducing the amount of antimicrobial required. For this reason, the application of moderate heat treatments and/or the preservation of the foodstuff in cold, refrigerated temperatures may play a key role. By using this method, a stable and, from a microbiological viewpoint, safe food can be produced without any loss in sensory quality. In this context various aroma compounds and citron essential oils containing citral, β-pinene, limonene, linalool, and α-pinene, combined with mild heat treatment, were used to inhibit the growth of *S. cerevisiae* in non-carbonated soft drinks (Belletti et al., 2007).

## Dairy products

Fresh dairy products are ready-to-eat foods easily contaminated by undesirable microorganisms. Some of them are spoilage microorganisms which may produce unwanted visual appearance and diminish the commercial value of cheese, other ones are pathogens that affect product safety. Moreover, fungal spoilage can also occur. Recently, some studies have recorded the efficacy of natural compounds, alone or in combination with other preservation methods, when directly applied to milk (Cava et al., 2007) or to cheese by spraying, immersing, or dusting the products. Antimicrobials may also be spread onto the packaging materials that come in contact with the cheese or incorporated into the plastic films used for packaging (Conte et al., 2007). A brief overview of some recent examples of natural active agents applied to cheese is reported.

The effectiveness of lysozyme and EDTA on microbiological shelf life of mozzarella cheese was studied by Sinigaglia et al. (2008). Mozzarella was packaged in a brine that contained lysozyme (0.25 mg mL^−1^) and different amounts of EDTA (10, 20, and 50 mmol L^−1^), and stored at 4 ± 1°C for 8 days. The packaging system significantly inhibited growth of coliforms and *Pseudomonadaceae*, without affecting the typical lactic acid bacteria. Conte et al. (2011) also evaluated the effects of lysozyme and EDTA in burrata cheese packaged under MAP (95:5 CO_2_:N_2_), thus demonstrating that these compounds were valid to prolong cheese shelf life, especially at high lysozyme concentrations.

Different release systems containing nisin and natamycin were also used in various works to create an additional hurdle for spoilage microorganisms in dairy products. For example, edible coatings made of galactomannans incorporating nisin were tested against *L. monocytogenes* in Ricotta cheese. The system not only help in retarding the growth of *L. monocytogenes* but also help in the maintenance of water content, therefore reducing cheese weight loss (Martins et al., 2010). Cao-Hoang et al. (2010) used sodium caseinate-based films for incorporating nisin to be active in mini red Babybel cheese. The active films affected *L. innocua* inoculated on cheese surface. As reported by Ture et al. (2011) natamycin was incorporated into wheat gluten and methyl cellulose biopolymers and tested against *A. niger* and *P. roquefortii* inoculated on surface of fresh kashar cheese. In a study conducted by Fajardo et al. (2010) natamycin was also used in combination with chitosan. In particular, the effects of the chitosan coating containing natamycin (0.50 mg/mL) on semi-hard cheese were assessed. The natamycin coated samples presented a decrease of moulds and yeasts compared to the control after 27 days of storage. The effects of nisin, natamycin, and their combination into a cellulose polymer matrix were also studied by dos Santos Pires et al. (2008). Films efficacy was first evaluated *in vitro* and then on sliced mozzarella cheese. Best effects were found when the two compounds were applied together on cheese.

As regards the applications of essential oils, numerous examples are reported in the literature (Table 2). The addition of different concentrations of lemon extract in the brine of mozzarella and in a gel solution made up of sodium alginate was evaluated by Conte et al. (2007). Shelf-life tests were run at 15°C to simulate thermal abuse. An increase in shelf-life of all active packaged mozzarella cheese was observed, confirming that the investigated substance may exert an inhibitory effect on the microorganisms responsible for spoilage, without affecting the typical dairy microbial population. As reported by Otaibi et al. (2008), three essential oils, thyme, marjoram and sage, were added to concentrated yoghurt (labneh) at concentrations of 0.2, 0.5, and 1.0 ppm. The better concentration of each essential oil was 0.2 ppm that allowed obtaining a shelf-life up to 21 days. Singh et al. (2011) also added essential oils in yogurt. The anise volatile oil and its oleoresin added to yogurt (prepared from buffalo's milk) (stored up to 20 days at 4 ± 1°C) at various concentrations (0.1–1.0 g L^−1^) were effective in controlling spoilage microorganisms. Eleven essential oils were evaluated *in vitro* for their antibacterial properties against *Vancomycin-resistant Enterococci* and *E. coli* O157:H7 (Selim, 2011). The most active essential oils against bacteria were thyme oil, eucalyptus, juniper, and clove oils. Furthermore, their effects were evaluated against the same microbial groups experimentally inoculated in Feta soft cheese and stored at 7°C for 14 days. The addition of thyme oil at concentrations of 0.5 and 1% caused a significant reduction in microbial growth. On Feta cheese inoculated with *E. coli* O157:H7 and *L. monocytogenes* oregano and thyme (0.1 or 0.2 and 0.1 ml/100 g) combined with MAP were also tested by Govaris et al. (2011). In the control Feta microbial strains survived up to 1 month of storage. On the contrary, in Feta cheese treated with oils a significant reduction of microbial growth was found. The use of chitosan, lemon, and sage extract in Fior di latte cheese was assessed by Gammariello et al. (2010). Different concentrations of active substances were added during processing cow's milk. Lemon extract and chitosan showed a good compromise between the antimicrobial effectiveness and the sensory impact caused by their addition, thus promoting a satisfactory shelf life increase (129%). In contrast, the addition of sage extract negatively affected the sensory properties, thus making the cheese unacceptable.

**Table 2:** **Relevant examples of natural active compounds applied to dairy products**.

**Products and storage conditions**	**Natural compounds**	**Main results**	**References**
West African soft cheese	Treatment with eucalyptus oil and lemongrass oil	The treatment of eucalyptus oil 75% plus 25% lemon grass exerted a positive impact on the nutritional, sensory, and microbial values of West African soft cheese	Belewu et al., 2012
Ricotta cheese stored under modified atmosphere at 4°C	Coating with a chitosan/whey protein edible film	The chitosan/whey protein film slowed detrimental phenomena. The viable numbers of lactic acid bacteria and mesophilic and psychrotrophic microorganisms were significantly lower in the chitosan/whey protein coated cheese, compared to the control	Di Pierro et al., 2011
Traditional Minas Serro cheese	Nisin	Nisin was effective in reducing *S. aureus* count in Serro cheese. A reduction of 1.2 and 2.0 log cycles in *S. aureus* count was observed from the 7th day of ripening for cheese containing 100 IU mL^−1^ and 500 IU mL^−1^ of nisin, respectively, compared with control sample	Pinto et al., 2011
Fresh cheese Tosèla	Antimicrobial compounds produced by six strains of non-starter lactic acid bacteria. In particular, *Lactobacillus paracasei* NdP78 was also found to produce a bacteriocin	Cheese showed higher concentrations of lactobacilli (7.90 log CFU/g) and streptococci (6.10 log CFU/g), lower development of coliforms and staphylococci than control cheese	Settanni et al., 2011
Caprese salad packaged under MAP (65% N_2_, 30% CO_2_, and 5% O_2_)	Dipping with thymol (400 ppm)	The combined use of thymol and MAP decreased the coliform populations from 5.65 to 4.23 log CFU/g and extended the microbiological shelf-life from 3.77 to 12 days. It also decreased the concentration of *Pseudomonadaceae*	Bevilacqua et al., 2007
Gorgonzola cheese	Natamycin-incorporated film in the production process of cheese	Films with 2 and 4% natamycin presented satisfactory results for *P. roquefortii* inhibition	de Oliveira et al., 2007

Pires et al. (2009) developed a microbial sachet incorporated with allyl-isothiocyanate. Its efficiency was tested against yeasts, molds, *Staphylococcus* sp. and psychrotrophic bacteria in sliced mozzarella cheese stored at 12°C ± 2°C. A reduction of 3.6 log cycles was observed in yeasts and molds counts in the mozzarella packed with the antimicrobial sachet over 15 days of storage time. The sachet also showed an antibacterial effect on *Staphylococcus* sp.; however, psychrotrophic bacteria were very resistant. A new dairy product “Karishcum” obtained by adding *Curcuma Longa* (Curcumin or Turmeric) to classic Karish cheese at a rate of 0.3% (w/v) was realized in a study conducted by Hosny et al. (2011). A primary experiment was done to determine the correct percentage of Curcumin addition to cheese milk to get a good taste and a long shelf life. The behavior of pathogenic bacteria in the artificially contaminated product during cold storage for 14 days, revealed that addition of the extract (0.3%) determined a reduction of bacterial counts of about 1 log of *S.* Typhimurium and two log of *P. aeurogenosa* and *E. coli* 0157:H7.

## Meat-based products

Spoilage of meat products contributes to deterioration of texture and change in flavor and color. The use of natural antibacterial compounds, such as extracts of spices and herbs, essential oils, organic acids, salts, and bacteriocins is reported in the literature to improve the shelf life of meat (Jamilah et al., 2008; Jałosńska and Wilczak, 2009). Some applications are reported in Table 3.

**Table 3:** **Relevant examples of natural active agents applied to meat-based products**.

**Products and storage conditions**	**Natural compounds**	**Main results**	**References**
Fresh minced beef patties packaged under MAP	Thymol (250, 500, 750 mg/Kg)	Better effects on product quality were obtained for sample with increased amount of thymol, under MAP conditions (shelf life about 7 days)	Del Nobile et al., 2009a
Minced beef mixed with soy-protein stored at 4°C	Sage essential oil (0.1, 0.3, and 0.5%)	The highest concentration of essential oil controlled development of main microorganisms	Ahmed and Ismail, 2010
Meat-balls stored at 10°C	0.2% of cranberry, rosemary, and lovage extracts	Rosemary extract was the most effective on product shelf life (13.3 days)	Jałosńska and Wilczak, 2009
Sausages stored at 4°C under vacuum conditions	Sodium lactate (0%, 0.6%, 1.2%, 1.8%) as alternative to nitrite	Sodium lactate improved the microbiological quality, extended shelf life, and exhibited a better antimicrobial effect than nitrite	Bingöl and Bostan, 2007
Fresh sausage	Oregano and marjoram essential oil	Addition of oregano and marjoram essential oil exerted a bacteriostatic effect	Busatta et al., 2007, 2008
Broiler chicken wings stored at 4°C	Dipping treatment for 10 min with chlorine dioxide, lactic acid, and fumaric acid	Samples treated with lactic acid alone showed the most effective reduction on *E. coli* and mesophylic bacteria	Hecer and Guldas, 2011
Fresh chicken meat stored under MAP at 4°C	Treatments with nisin and EDTA, alone or in combination	Chicken was better preserved under treatments with 500 IU/g of nisin and 50 mM of EDTA, even up to 24 days	Economou et al., 2009
Fresh beef	Organic acids (citric, lactic, acetic, and tartaric)	Organic acids promoted a significant shelf life extension	Jamilah et al., 2008
Fresh chicken sausage stored at 4°C	Rosemary or Chinese mahogany (500, 1000, 1500 ppm)	Chinese mahogany and rosemary improved meat quality	Liu et al., 2009
Turkey-bologna stored at 4°C	Coating with gelatin containing Nisaplin and Guardian	Both Nisaplin film and Guardian film effectively inhibited *L. monocytogenes*	Min et al., 2010
Meat pieces	Combined application of oregano essential oil and acetic acid	Combination of essential oils and organic acids inhibited microbial growth and proliferation of pathogens such as *S. aureus*	de Souzaetal., 2009

Mastromatteo et al. (2011b) suggested the combined use of natural antimicrobials, such as lemon and thymol, and MAP to improve the shelf life of reduced pork back-fat content sausages. In particular, the application of thymol and thymol-MAP limited the development of *Pseudomonas* spp., responsible for sausages' unacceptability. The use of bay essential oil combined to MAP without oxygen (20% CO_2_—80%N_2_) was suggested to control *L. monocytogenes* and *E. coli* growth and also to extend the shelf life of naturally contaminated ground chicken meat (Irkin and Esmer, 2010). Moreover, the addition of essential oils of marjoram and rosemary to beef patties formulated with mechanically deboned poultry meat at a concentration of 200 mg/kg reduced lipid oxidation and improved the sensory characteristics (Mohamed and Mansour, 2012). To reduce microbial growth and to preserve the oxidative stability of mortadella, a Bologna-type sausage, the addition of orange dietary fiber (1%), rosemary essential oil (0.02%) and thyme essential oil (0.02%), combined with specific storage conditions, showed very desirable effects (Viuda-Martos et al., 2010, 2011). Antioxidant and antibacterial effects of rosemary, orange, and lemon extracts was also investigated in cooked Swedish-style meat-balls. Results indicated that significant advantages were obtained using rosemary and citrus extracts in rancidity-susceptible meat products; however, only rosemary slightly reduced lactic acid bacteria (Fernàndez-Lòpez et al., 2005). Ayachi et al. (2007) reported that the addition of a mixture of organic acids (sodium lactate 90% and sodium acetate 10%) at different concentrations (from 0 to 20 g/Kg) on Marguez sausages, made with lamb and beef, significantly reduced microbial cell loads during storage at 8°C.

Chitosan (0.5% and 1%) added individually or in combination with nitrites (150 ppm) as ingredients was tested to protect fresh pork sausages from microbial spoilage. Its application as active coating was demonstrated (Bostan and Isin-Mahan, 2011). Soultos et al. (2008) found chitosan active against total viable count, lactic acid bacteria, *Pseudomonas* spp., *B. thermosphacta, Enterobacteriaceae*, yeasts, and moulds.

Krisch et al. (2010) compared the antimicrobial effect of commercial herbs, spices and essential oils (fresh and dried garlic, onion, thyme, marjoram, and oregano) in minced pork. While fresh spices showed weak or no inhibition on viable cells of minced pork, some effects of essential oils were observed. Best shelf life values were obtained for pork meat added with garlic and marjoram oil. Dipping of thyme and oregano oil in concentrations of 0.1 and 0.3% were carried out to improve the shelf life of meat-based products (Karabagias et al., 2011). The combination of dipping and MAP extended the shelf life to about 22 days, against 7 days of the control sample packaged in air.

A relevant preservation effect for fresh chicken breast meat, stored at 4°C, was obtained by dipping meat in oregano oil, prior to packaging under MAP (Chouliara et al., 2007). Fratianni et al. (2010) also proposed use of thyme and balm essential oils to decrease the natural microflora of chicken breast meat. In particular, balm essential oil significantly limited growth of *Salmonella* sp., whereas thyme essential oil effectively inhibited growth of *E. coli*. It was also widely demonstrated that dipping with lactic acid, clove oil and vitamin C can exert significant advantages over dipping with lactic acid alone to improve shelf life of buffalo meat steaks. In particular, use of clove oil along with lactic acid provided synergistic antioxidant and antimicrobial effects; the inclusion of vitamin C also stabilized product color (Naveena et al., 2006). Ntzimani et al. (2010) highlighted that combined use of EDTA, lysozyme, rosemary, and oregano oil extended shelf life of semi-cooked coated chicken fillets stored under vacuum packaging at 4°C to more than 2 weeks.

Effects of pork chops dipped in organic acids, such as ascorbic acid (500 ppm) and citric acid (250 ppm) individually or in combination, packaged under MAP and vacuum and stored at 1°C were studied by Huang et al. (2005). Ascorbic acid dipping reduced psychrotrophic microbial count, while ascorbic and citric acids improved lipid stability. The obtained results were enhanced by packaging under MAP conditions.

As regards bacteriocins, the combined use of lactoferrin and nisin on naturally contaminated Turkish-style meat-balls was proposed. Treatment with lactoferrin alone and in combination with nisin significantly reduced spoilage bacterial counts (total aerobic bacteria, coliform, *E. coli*, total psychrophilic bacteria, *Pseudomonas* spp., yeast and molds) and extended shelf life to 10 days (Colak et al., 2008). The synergistic antimicrobial activity of lysozyme, nisin, and EDTA against *L. monocytogenes* and meat-borne spoilage bacteria in ostrich patties packaged in air and vacuum was observed by Mastromatteo et al. (2010a). In particular, the antimicrobial treatment was effective for controlling growth of lactic acid bacteria even if it was not effective against Gram-negative bacteria.

## Fish-based products

Fresh fish is a highly perishable product due to its biological composition. The main cause of deterioration is the activity of spoilage seafood microorganisms that provoke loss of essential fatty acids, fat-soluble vitamins and protein functionality, production of biogenic amines, and formation of off-odors (Gram and Dalgaard, 2002).

Literature widely demonstrates that treatments with natural compounds are effective preservation methods for fish products (Table 4). Different effects are generally exerted depending on the active agent used and on the characteristics of the raw material. Some examples are provided hereinafter. Erkan et al. (2011) proposed the use of thyme (1%) and laurel essential oil (1%) to extend the shelf life of bluefish by about 3–4 days. The quality of hot smoked rainbow trout packaged under vacuum and treated with thymol and garlic oil (1%) was also improved (Erkan, 2012). Kykkidou et al. (2009) demonstrated that combination of MAP and thyme essential oil (0.1%) resulted in a significant shelf life extension of fresh Mediterranean swordfish fillets. In particular, the addition of thyme essential oil extended product shelf life (13 days) if compared to the control (8 days), whereas its combination to MAP conditions further prolonged product shelf life (about 20.5 days). Goulas and Kontominas (2007) also showed that oregano essential oil (0.8 %) extended shelf life of sea bream fillet by more than 17 days. Similar results were also reported by Pyrgotou et al. (2010) on rainbow trout where the similar combined strategies reduced the cell load of main spoilage microorganisms. Corbo et al. (2008) highlighted the possibility to extend the microbial acceptability limit of fresh fish burgers by using a mix of thymol, grapefruit seed extract, and lemon extract. The mixture of the three natural compounds prolonged the sensory quality without compromising the flavor of fish. Each antimicrobial compound was first tested *in vitro* against the main fish spoilage microorganisms (Corbo et al., 2009). In a subsequent work, thymol, grapefruit seed extract, and lemon extract were used in combination to MAP to demonstrate that MAP further enhanced the effects of the natural active compounds. (Del Nobile et al., 2009b). Min et al. (2009) demonstrated the antimicrobial and antioxidant activity of purple rice bran extract against catfish patties.

**Table 4:** **Relevant examples of natural active agents applied to fish-based products**.

**Products and storage conditions**	**Natural compounds**	**Results**	**References**
Rainbow trout fillets packaged under vacuum	Oregano essential oil (0.2, 0.4%)	The combination of oregano (0.2%) and vacuum resulted in a significant shelf life extension of trout fillets (11–12 days) if compared to the control packaged in air (5 days)	Frangos et al., 2010
Rainbow trout fillets packaged with oxygen absorber	Oregano essential oil (0.4%)	The antimicrobial compound improved the sensory shelf life	Mexis et al., 2009
Fish burgers packaged under vacuum	Rosemary extract 0.4% and 0.8%	Rosemary extract, in combination with vacuum packaging was effective in controlling microbial growth and biochemical changes	Uçak et al., 2011
Fried mullet fish fillets	Edible coating solution mixed with thyme (2.5, 5%) and marjoram (2.5, 5%)	Thyme and marjoram have strong effects against Enterobacteriaceae	Yasin and Abou-Taleb, 2007
Sea bass fillets packaged under different MAP	Thyme essential oil (0.2%)	Essential oil improve the quality of sea bass fillets when used in combination with 60% CO_2_-30% N_2_-10% O_2_, providing a shelf life of 17 days as compared to 6 days of the control samples	Kostaki et al., 2009
Cooked blue swimming crab meat	Sodium acetate dipping treatments (1, 1.5, and 2%)	Shelf life of product dipped in 2% for 2 min was 12 days compared to the control (6 days)	Lohalaksanadech and Sujarit, 2011
Peeled shrimps packaged under MAP	Coating with thymol (500, 1000, 1500 ppm)	Shelf life of about 14 days for the active coating (1000 ppm) packaged under MAP compared to the sample in air (5 days) was obtained.	Mastromatteo et al., 2010b

Coatings enriched with essential oils were also proposed in literature as valid technique to improve quality of fish products. As example, Ojagh et al. (2010) reported that the use of a coating with chitosan and cinnamon essential oil improved trout fillet shelf life (16 days vs. 10 days of the control) and in particular it enhanced texture, odor, and color. Similar results were also obtained for trout fresh fillets coated with gelatin enriched with cinnamon oil (1%, 1.5%, and 2%). In particular, experimental data indicated that the active coating can be suitable for preserving the fillets and maintain quality to an acceptable level (Andevari and Rezaei, 2011).

To control quality of northern snakehead fish fillets at refrigeration temperature cinnamon, coatings with nisin and EDTA, alone and in combination were used (Lu et al., 2010).

Acetic acid, glucono-delta-lactone and chitosan were tested, individually and in combination, to inhibit microbial growth in surimi. The results showed that microbial proliferation was successfully inhibited by packaging the fish ball in 1% chitosan dissolved in 1% of acetic acid (Kok and Park, 2007). Shirazinejad et al. (2010) evaluated the use of lactic acid alone and in combination with nisin for reducing microorganisms on chilled shrimp. Best results against *Pseudomonas spp*. were obtained for samples treated with the mixture of lactic acid and nisin.

## Cereal-based products

Shelf life of bread is generally limited due to the staling phenomenon and fungi spoilage, in particular moulds. Among strategies aimed to improve quality of bread, some effects were reported by using different natural compounds. In particular, it was reported that chitosan coating improved bread quality by inhibiting microbial growth and retarding oxidation and staling. A reduced microbial proliferation was obtained for bread coated with chitosan during storage at room temperature (No et al., 2007).

Rehman et al. (2007) reported different applications of citrus peel essential oils in bread. Results demonstrated that the oils influenced sensory characteristics and delayed microbial growth. Maximum inhibitory effect against moulds and bacteria was achieved by spraying peel essential oil. The combination of MAP and mustard oil was proposed for wheat and rye bread artificially inoculated with moulds (Suhur and Nielsen, 2005).

Breads prepared from wheat flour by adding different additives were also evaluated by Latif et al. (2005). The studies on colony count in bread at different storage time showed that treatment containing 0.32% of suhanjna, 3% of lecithin, and 0.1% of ascorbic acid proved to be most effective. The different combination of the three selected natural additives improved bread shelf life. In particular, lower cell loads of yeasts and moulds were observed for bread with lecithin and ascorbic acid (Latif and Masud, 2006). An active packaging with cinnamon essential oil combined with MAP was tested to increase the shelf life of gluten-free sliced bread. Results showed that the active packaging is better than MAP to increase product shelf life because it inhibited microbial growth while maintaining the sensory properties of the gluten-free bread (Gutiérrez et al., 2011).

Natural active compounds were also applied to fresh pasta that is a product easily perishable for its high water content. Del Nobile et al. (2009c) proposed the use of different natural antimicrobial compounds such as thymol, lemon extract, chitosan, and grapefruit seed extract at different concentrations (2000 mg/kg and 4000 mg/kg) to improve the microbiological stability of refrigerated amaranth-based fresh pasta. Results pointed out that chitosan were the most successful among the investigated compounds in slowing down the spoilage, whereas lemon extract was the less effective. In a subsequent work, the antimicrobial activity of chitosan in combination with different MAP was tested. It was found that among the tested MAP conditions, the combination of 30:70 N_2_:CO_2_ extended the shelf life beyond two months (Del Nobile et al., 2009d). The antimicrobial activity of chitosan against the main microorganisms of fresh pasta was also reported by Costa et al. (2010). In particular, statistically significant differences were found between the shelf life of pasta with chitosan packaged under MAP conditions in a low barrier film made up of polypropylene and in a multilayer high barrier film made up of polyethylene terephthalate, ethylene-vinyl alcohol, and polyethylene.

To improve the shelf life of yellow alkaline noodle Rosyid et al. (2011) tested the antibacterial activity of ethanol and water extracts of six types of leaves against the principal spoilage microbial groups of this product. Results highlighted that ethanol extracts of aromatic leaf, *Murraya koenigii* L., added in yellow alkaline noodle contributed to improve shelf life better than the other extracts. Budka and Khan (2010) demonstrated that essential oils from basil, thyme and oregano exhibit bactericidal properties against *B. cereus* in rice-based foods. The antioxidant and antimicrobial activity of different natural compounds (anise, black cumin, rosemary and sage) were also tested to increase shelf life of some bakery products. Preliminary results showed that both Gram-positive and Gram-negative bacteria were sensitive to all tested essential oils and phenolic compounds (Basuny et al., 2012).

## Concluding remarks and future perspectives

Most food products require protection against microbial spoilage during storage. Consumers demand safe natural products. This drives the search of food authorities and researchers for mild preservation techniques to improve microbial quality and safety without causing nutritional and organoleptic losses. In this context natural compounds are gaining a great interest from research and industry, due to the potential to provide quality and safety benefits, with a reduced impact on human health. In addition, utilization of natural active agents promotes the accepted criteria of food sustainability. The numerous experimental applications of essential oils, enzymes, bacteriocins, chitosans, and organic acids to various fresh perishable foods demonstrate that they are well suited to be utilized as preservatives in foods and could be often valid alternatives to synthetic food additives. However, there is a need to search for new sources of antimicrobial substances, including plant metabolites. Natural products have been the most successful source of drugs ever. Historically, the most important natural sources have been plants. Research progressed along two major lines: ethnopharmacology (medicinal herbs, substances of abuse, ordeal poisons) and toxicology (poisonous plants, venomous animals, arrow, and fish poisons). These strategies have produced many valuable drugs and are likely to continue to produce lead compounds. It must be stated that traditional medicines have not been found by systematic research but by a combination of coincidence and observation, and at best by trial and error. In order to further promote the application of natural active compounds at industrial level, some factors are of striking importance. First of all it is necessary to have a good understanding of the mechanism by which antibacterial agents operate. For many natural compounds these information are still lacking. Better understanding of the modes by which antimicrobials can control microorganisms should provide solid grounds for engineering new and upgraded derivatives with optimized potency and stability. Further research is still necessary for specific case-study because it is well demonstrated that the combination of more than one active agent not always amplifies the antimicrobial effects. Very often, the combined use of some natural essential oils did not induce synergistic effects. So, generally specking some considerations must be taken into account before using antimicrobials in food preservation. One of them is the possible existence of interactions between compounds and food components. Moreover, also the adoption of active compounds under MAP conditions could exert different effects depending on the product.

In the specific case of essential oils, despite their great potential, their use in food preservation remains limited mainly due to their intense aroma and toxicity problems. Several authors have reported changes in the organoleptic properties of the food when these oils are used. To minimize the required doses and improve the effectiveness of active coatings enriched with essential oils, interesting options would be micro- and nanoencapsulation of active compounds. In addition, the use of combinations of different food preservation systems, such as the use of proper temperature, could represent another solution to the above-mentioned problem. As regards toxicity, the ingestion of high doses of essential oils can induce serious problems. Thus, it is necessary to find a balance between the effective compound dose and the risk of toxicity. It is also worth noting that the use of essential oils remains expensive, so from an economic point of view this preservation strategy needs further enhancement. Moreover, more specific ISO standards are also necessary to assess the legal aspects to set out the definition, the general rules for their use, the requirements for labeling and the maximum levels authorized.

### Conflict of interest statement

The authors declare that the research was conducted in the absence of any commercial or financial relationships that could be construed as a potential conflict of interest.
